# Donning Sterile Surgical Gloves – A Prospective Clinical Audit of Young Surgeons at a Tertiary Care Hospital of Lahore, Pakistan

**DOI:** 10.7759/cureus.32831

**Published:** 2022-12-22

**Authors:** Rana Arslan Rabbani, Muhammad Khalil-ur-Rehman, Fahad Hussain, Shah Ahmad Fazli, Haseeb Mehmood Qadri, Nida Manzoor, Menahil F Cheema, Amjid ul Haq, Fiza Ismail, Junaid Saffi

**Affiliations:** 1 Surgery, Lahore General Hospital, Lahore, PAK; 2 Surgery, District Headquarter Hospital Okara, Okara, PAK; 3 Surgery, Sharif Medical and Dental College, Lahore, PAK

**Keywords:** surgical site infection, world health organization, surgical scrubbing, surgical glove, hygiene, clinical audit, asepsis

## Abstract

Background

Sterilization and aseptic surgical techniques are the most important keys to successful postoperative outcomes. The standard surgical gloving technique causes early wound healing and reduces morbidity and mortality.

Objective

To assess the standard technique of donning sterile surgical gloves while scrubbing among young surgeons.

Material and Methods

This two-week prospective audit involved 60 young residents and house officers after ensuring ethical implications. Participants were observed unannounced for donning sterile surgical gloves in the surgical operation theatre (OT) according to the standard criteria set by World Health Organization (WHO) before and after the relevant intervention. The intervention was made through a clinical lecture, live demonstration, and hands-on sessions. After a detailed literature study, a pro forma was generated to record participants' compliance with 14 steps of donning sterile surgical gloves. Data was sent to a statistician for descriptive analysis.

Results

About 72.14% of the participants followed the standard criteria of donning sterile surgical gloves before intervention. This percentage raised to 90.71% after the intervention, showing marked improvement.

Conclusion

Pre-intervention and post-intervention observations showed apparent differences in compliance rates for the standard criteria of donning sterile surgical gloves. This scientific study signifies the need for such clinical audits to boost standard surgical practices, especially among newcomers.

## Introduction

One of the major concerns of the perioperative team is surgical site infections (SSIs). An infection that develops due to the transfer of microorganisms to the patient's wound during surgery is called SSI [1]. It threatens the lives of millions of people each year due to antibiotic-resistant infections. It is the most common type of nosocomial infection in patients who have undergone surgical procedures [2]. Approximately seven percent of the patients in developed nations and more than 25% of the patients in developing countries are affected by healthcare-associated infections (HCAIs) [3]. SSIs result in delayed wound healing, increased hospital stays, increased use of antibiotics, unnecessary pain, and in extreme cases, even death of the patient. Hence, their prevention is a crucial aim of health services. According to estimates, 26-54% of such infections can be avoided. Proper aseptic techniques, including hand hygiene, can prevent these infections. The surgical team should wear a sterile gown and gloves to reduce the risk of SSIs [2,3].

Aseptic practices, including scrubbing, gloving, barrier clothing, drapes, and instrument sterilization, are pivotal to protecting the integrity of the sterile field [4]. However, because of the fast-paced nature of the operating room (OR), the short time available for training, and the scarcity of experienced medical personnel, these abilities are challenging for medical students to learn [5]. Hand preparation for surgery includes initial hand washing, procedural steps of rubbing, drying off hands, wearing an operating gown, and wearing sterile surgical gloves [6]. In 2008, an initiative of 'Clean Care is Safer Care' was launched by the World Health Organization (WHO) to improve hand hygiene (HH) compliance among healthcare workers (HCWs) [3].

Gloves, gowns, and masks have a role in preventing infections but are often misused, increasing service costs unnecessarily [7]. This surgical audit aims to assess glove-donning practices in the elective surgical operation theater of Lahore General Hospital, Lahore. There is a scarcity of such clinical audits on standard donning sterile surgical gloves practices in the existing English scientific literature. To our best knowledge, this is the first in-field prospective audit on this topic from Pakistan, using the WHO standard guidelines and involving education and assessment of the participants.

## Materials and methods

After approval from the Ethical Review Board of Lahore General Hospital, approval number SU-III/10/22/LGH, this two-week clinical audit was carried out at the elective operating room (OR) of the Department of General Surgery, Unit-II at Lahore General Hospital from January 17, 2022, to January 31, 2022. We have two days dedicated to elective surgeries each week. The ward's 60 surgical trainees and house officers were part of the study. An elaborate literature study was done to develop this research's plan, design, and implications.

Pre-intervention observation

In the third week of January, all young surgeons were observed while donning surgical gloves before routine preparation for elective surgery on two different days. This observation was noted and compared by our audit team of six authors with the standard guidelines set by WHO on donning sterile surgical gloves [8]. We used the following judgment criteria to evaluate all young surgeons (Table 1). Given in the table, each 'yes' represents 1 correct answer, and each 'no' represents 1 wrong answer from a total of 14 [8]. For each participant, we calculated compliance with these 14 criteria and summed the score of all participants to each criterion individually. Percentages were used to determine the overall compliance of all participants to each criterion separately. 

**Table 1:** Guidelines on Donning of Sterile Surgical Gloves by the World Health Organization (WHO)

Criteria	Description	Response	
1	Perform hand hygiene before an "aseptic procedure" by hand rubbing or hand washing.	Yes	No
2	Check the package for integrity. Open the first non-sterile packaging by peeling it completely off the heat seal to expose the second sterile wrapper, but without touching it.	Yes	No
3	Place the second sterile package on a clean, dry surface without touching the surface. Open the package and fold it towards the bottom to unfold the paper and keep it open.	Yes	No
4	Using the thumb and index finger of one hand, carefully grasp the folded cuff edge of the glove.	Yes	No
5	Slip the other hand into the glove in a single movement, keeping the folded cuff at the wrist level.	Yes	No
6, 7	Pick up the second glove by sliding the fingers of the gloved hand underneath the cuff of the glove.	Yes	No
		Yes	No
8	In a single movement, slip the second glove onto the ungloved hand while avoiding any contact/resting of the gloved hand on surfaces other than the glove to be donned (contact/resting constitutes a lack of asepsis and requires a change of glove).	Yes	No
9		Yes	No
10		Yes	No
11	If necessary, after donning both gloves, adjust the fingers and interdigital spaces until the gloves fit comfortably.	Yes	No
12, 13	Unfold the cuff of the first gloved hand by gently slipping the fingers of the other hand inside the fold, making sure to avoid any contact with a surface other than the outer surface of the glove (lack of asepsis requiring a change of gloves).	Yes	No
		Yes	No
14	The hands are gloved and must touch exclusively sterile devices or the previously disinfected patient's body area.	Yes	No

The intervention

After the first round of observation and data collection, all participants were given a presentation that included a video demonstration of the standard surgical gloving technique and a WHO-based pamphlet on hand hygiene. Subsequently, a consultant surgeon also gave an individual demonstration in the operation theatre to the trainees and house surgeons. A pictorial guide was also displayed inside each of the six operation theatres.

Post-intervention assessment

In the second phase of the audit, i.e., the last week of January, all participants were observed again for their compliance against the standard surgical gloving criteria. Each step followed successfully was awarded 1 mark, and all 14 steps were evaluated individually. Everyone was assessed with a total score of 14 (1 step = 1 mark), and all the compliance scores were calculated individually and collectively to compute the average improvement. For each participant, we calculated compliance with these 14 criteria and summed the score of all participants to each criterion individually. Percentages were used to determine the overall compliance of all participants to each criterion separately. Compiled data was sent to a statistician. Using the Statistical Package for Social Sciences (SPSS) version 26.0, frequencies and mean values were calculated and analyzed to identify individual and group compliance rates before and after the intervention. At the end of this audit, we also took feedback from our participants regarding the intervention they thought was the best in helping them learn the standard practice of donning sterile surgical gloves.

## Results

Sixty surgical trainees and house officers, 44 males and 16 females, were monitored for their awareness and application of standard hand hygiene skills and donning sterile surgical gloves. As determined by cumulative percentages of compliance of all participants to each criterion separately, criteria 1, 13, and 14 saw no change in their pre-intervention and post-intervention compliance rates because these steps were followed aptly by all participants. Criterion 11, adjusting gloves concerning interdigital spaces, was missed by 10% of candidates pre-intervention and post-intervention. The greatest rate of improvement was seen in criterion 8, where 55% of participants learned to slip the second glove onto the ungloved hand in a single movement, with a difference of 55% pre-intervention and post-intervention. Overall, all participants met 72.14% of the standard criteria during pre-intervention observation, while 90.71% met them after the intervention. The percentage improvement in pre-intervention and post-intervention compliance rates of donning sterile surgical gloves remains remarkable, i.e. 18.57%. After this audit, there was a considerable improvement in compliance with all steps of the gloving technique (table 2).

**Table 2:** Pre-Intervention & Post-Intervention Compliance Percentage Improvement for Various Criteria.

Criteria	Compliance/Adherence Rates (%)		% Improvement
	Pre-intervention	Post-intervention	
1	100	100	-
2	90	95	5
3	85	100	15
4	70	85	15
5	55	95	40
6	55	90	35
7	55	85	30
8	35	90	55
9	45	80	35
10	65	85	20
11	90	90	-
12	65	75	10
13	100	100	-
14	100	100	-
Mean Compliance	72.14	90.71	18.57

**Figure 1:**
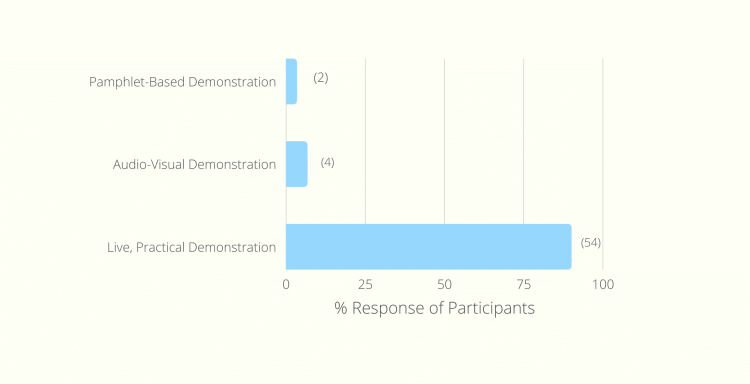
Best Intervention Method According to Participants.

When asked about the best intervention which helped the participants grasp the standard technique of donning sterile surgical gloves, 90% of participants (n=54) voted for "live, practical demonstration" as the best method to teach the technique, as demonstrated in Figure 1.

## Discussion

Surgical wounds can be clean, clean-contaminated, contaminated, and dirty. Appropriate sterile surgical glove donning is a requisite for asepsis and early wound healing. Though skill memory is a blessing, the process of learning from seniors, good or bad, is inadvertent. Reaching a level of perfection in skills requires the correct instruction and practice. According to Miller's pyramid, medical students acquire any talent through time [9].

In our study, the surgical trainees were utterly compliant with criteria 1, 11, 13, and 14. A scientific article published in 2022 in Nepal shows a mean compliance of 89% for criteria 4 and 5. The current study at Lahore General Hospital shows a compliance rate of 70% and 55%, respectively, before the intervention. The compliance with criteria 6 and 7 in the former study was 81% in contrast with a compliance rate of 55% in our study for both these steps [2].

The most significant improvement was seen with the adherence to criteria 8, in which, the adherence was 35% which rose to 90% post-intervention. Similarly, compliance with criteria 9 and 10 improved after the audit. Our participants showed adherence of 35%, 45%, and 65% for criteria 8, 9, and 10, in contrast to a better compliance rate, i.e., 74.4% shown by the health personnel at the operation theatre of the teaching hospital, Bharatpur. The latter was approximately 99% compliant with criteria 14. However, the young surgeons of our ward were 100% compliant with it [2].

Around 46 studies conducted by various scientists worldwide concluded that after the intervention, Hand Hygiene (HH) compliance improved, ranging from 1% to 66%, with the mean net improvement being an increase of 26% [3]. While in our study, a mean improvement of 18.5% was noted. For the intervention group in the study by Clancy et al., a mean of 67% was calculated as the overall post-intervention compliance rate. But, our study inferred a mean compliance of 90.71% for the post-intervention group [3].

The Bharatpur study showed an overall mean compliance score of 88.88%, while our study showed a mean compliance of 72.14% before intervention. Going one step further, we arranged a class to re-explain the WHO steps of donning sterile surgical gloves for young surgeons; following that, we did a re-audit which showed mean compliance of 90.71% after the intervention [2]. A study in Indonesia to evaluate adherence to hand hygiene and gowning showed that about 83.12% of surgical residents followed the protocols [6]. In the same study, the step with the lowest mean score was removing the gloves' wrapping by hand from within the gown's sleeves (criteria 2 in our study), while in our study, criteria 8 had the lowest mean [6].

An article published in India followed a 3-month baseline measurement period with a 4-month intervention period. Then an average of 17.2 months of follow-up measurements highlighted that the peak HH compliance was 90% at the 2-year follow-up. However, by the 3-year follow-up, it had dropped to 82%, indicating the continuous need for repeated audits [3]. Factors affecting the HI included religious beliefs, habituation to HH, staff workload, availability of hand hygiene, material, and the quality of hand hygiene feedback [3]. At the same time, we observed that most young surgeons needed to be aware of some steps. Lack of motivation, carelessness and arduous duty hours were a few reasons for decreased compliance.

We previously performed a study in 2021 on hand washing techniques using the same method, and we inferred that continuous education via videos, positive feedback, and guidance of house officers and medical residents improved the pre-intervention compliance of 63% to a remarkable 90.33% post-intervention [10].

We acknowledge the potential limitations of this study. The fact that it is a single-center audit with a small sample size is a limiting factor. We could not find adequate scientific literature about the nature of this study; hence productive comparisons could not be made.

## Conclusions

This clinical audit was performed for the first time, indicating that more audits are required to improve patient safety. This study examined surgeons' pre-intervention and post-intervention attitudes regarding the standard sterile surgical glove technique according to the WHO guidelines. This study found that both before and after the intervention, there were substantial shifts in the variables. Even though many surgeons have done their best to adhere to the WHO guidelines on surgical gloving techniques, many still need to meet expectations. On the other hand, significant progress was made through the training sessions. As a result, clinical audits and research that are conducted periodically are of the utmost significance in bringing about a good change in clinical practice.

Based on the mentioned data, more audits should be done on a regular basis to improve adherence to standard guidelines. This can lead to a significant improvement in the reduction of health hazards, which still, in turn, can help a country's economy by reducing prolonged hospital stays and minimizing the iatrogenic disease burden. Such clinical audits provide the framework to improve patient care collaboratively and systematically and educate healthcare professionals to become better caregivers and overcome their shortcomings. This also highlights the importance of intervention for amelioration.

## Appendices

List of Abbreviations

SSI: Surgical Site Infections

HCAI: Health Care Associated Infections

OR: Operating Room

WHO: World Health Organization

HH: Hand Hygiene

HCW: Healthcare Workers

SPSS: Statistical Package for Social Sciences

Authors' Contributions

RAR: Literature Study, Data Collection, Manuscript Writing & Drafting, Result Analysis and Manuscript Proof-reading;

MKR: Concept & Study Design, Literature Search and Supervision;

FH: Literature Study, Data Collection, Manuscript Writing & Writing & Drafting, Result Analysis, Figure Support and Manuscript Proof-reading;

SAF: Manuscript Writing & Drafting, Formatting; 

HMQ: Literature Search, Literature Study, Manuscript Writing & Drafting, Result Analysis and Interpretation, Figure Support, Manuscript Proof-reading, Formatting and Editing;

NM: Manuscript Writing & Drafting and Formatting;

MFC: Data Collection and Manuscript Writing & Drafting;

AH: Data Collection and Manuscript Writing & Drafting;

FI: Data Collection, Manuscript Writing & Drafting and Manuscript Proof-reading;

JS: Data Collection, Result Analysis and Manuscript Writing & Drafting, 

## References

[1] Tanner J, Dumville JC, Norman G, Fortnam M (2016). Surgical hand antisepsis to reduce surgical site infection. Cochrane Database Syst Rev.

[2] Biddhya KC, Bista B. (2022). Practice of gowning and gloving technique among health personnel at operation theatre of teaching hospital, Bharatpur. J Chitwan Med Coll.

[3] Clancy C, Delungahawatta T, Dunne CP (2021). Hand-hygiene-related clinical trials reported between 2014 and 2020: a comprehensive systematic review. J Hosp Infect.

[4] Jeyakumar A, Sabu S, Segeran F (2017). Adequacy of scrubbing, gowning and gloving among operating room nurses. IOSR-JNHS.

[5] Canton S, Foley C, Donnellan N. (2020). Development of surgical scrubbing, gowning and gloving checklist using the Delphi method. MedEdPublish.

[6] Handaya AY, Werdana VA (2019). Adherence to preoperative hand hygiene and sterile gowning technique among consultant surgeons, surgical residents, and nurses: a pilot study at an academic medical center in Indonesia. Patient Saf Surg.

[7] Saloojee H, Steenhoff A (2001). The health professional's role in preventing nosocomial infections. Postgrad Med J.

[8] (2009). WHO Guidelines on Hand Hygiene in Health Care: First Global Patient Safety Challenge Clean Care Is Safer Care. Geneva: World Health Organization. https://www.who.int/publications/i/item/9789241597906.

[9] J. Kasula J, Yerroju K. (2019). Skill of donning surgical gloves amongst residents: a neglected skill. Int J Surg.

[10] Hussain F, ur Rahman MK, Qadri HM, Rabbani RA (2022). Surgical hand washing-a clinical audit of young surgeons at a tertiary care hospital. Pakistan J Medical Health Sci.

